# Mistaken identity may explain why male sea snakes (*Aipysurus laevis*, Elapidae, Hydrophiinae) “attack” scuba divers

**DOI:** 10.1038/s41598-021-94728-x

**Published:** 2021-08-19

**Authors:** Tim P. Lynch, Ross A. Alford, Richard Shine

**Affiliations:** 1 CSIRO, Castray Esplanade, Hobart, TAS 7000 Australia; 2 College of Science and Engineering, James Cook University, Townsville, QLD 4811 Australia; 3 Department of Biological Sciences, Macquarie University, North Ryde, NSW 2109 Australia

**Keywords:** Behavioural ecology, Tropical ecology

## Abstract

Scuba-divers on tropical coral-reefs often report unprovoked “attacks” by highly venomous Olive sea snakes (*Aipysurus laevis*). Snakes swim directly towards divers, sometimes wrapping coils around the diver’s limbs and biting. Based on a focal animal observation study of free-ranging Olive sea snakes in the southern Great Barrier Reef, we suggest that these “attacks” are misdirected courtship responses. Approaches to divers were most common during the breeding season (winter) and were by males rather than by female snakes. Males also made repeated approaches, spent more time with the diver, and exhibited behaviours (such as coiling around a limb) also seen during courtship. Agitated rapid approaches by males, easily interpreted as “attacks”, often occurred after a courting male lost contact with a female he was pursuing, after interactions between rival males, or when a diver tried to flee from a male. These patterns suggest that “attacks” by sea snakes on humans result from mistaken identity during sexual interactions. Rapid approaches by females occurred when they were being chased by males. Divers that flee from snakes may inadvertently mimic the responses of female snakes to courtship, encouraging males to give chase. To prevent escalation of encounters, divers should keep still and avoid retaliation.

## Introduction

Diverse and abundant in tropical marine waters, sea snakes of the subfamily Hydrophiinae are secondarily aquatic taxa derived from terrestrial elapid (front-fanged) snakes^1,2^. Laboratory studies suggest that the venoms of some species of sea snakes are highly toxic^3^; and worldwide, bites by sea snakes kill many people annually^4,5^. Although those fatalities generally involve fishermen rather than recreational or other users of the ocean^6^, SCUBA divers frequently report unprovoked “attacks” which can imperil divers through inducing panic, even if the snake does not deliver a bite^7^.

As described by Heatwole^8^, these “attacks” involve rapid jerky zigzag movements, easily distinguished from the leisurely swimming mode of curious snakes. Understanding the causes for such “attacks” is of interest from two perspectives. First, why would a free-ranging snake approach and bite a person that has not harassed it, is too large to be a prey item, and could readily be evaded in the complex three-dimensional world of a coral reef? Extensive studies on terrestrial snakes (including elapids) show that these animals would far prefer to escape than to confront an approaching human^9^, so why should sea snakes be so different? Second, understanding the context of these approaches might suggest how divers should respond to rapid approach by a potentially lethal snake. This may be of practical value to recreational dive and eco-tourism operators as well as commercial divers to mitigate this common human-wildlife interaction.

Anecdotal reports suggest that Olive sea snakes (*Aipysurus laevis*) are the most frequent “attackers”, and that this behaviour is manifested most frequently during the snake’s breeding season^8,10–13^. To explore those ideas, we quantified the seasonal timing and identity of “attacking” snakes in the course of a 27-month scuba-based behavioural study of sea snake ecology.

## Results

**Figure 1:**
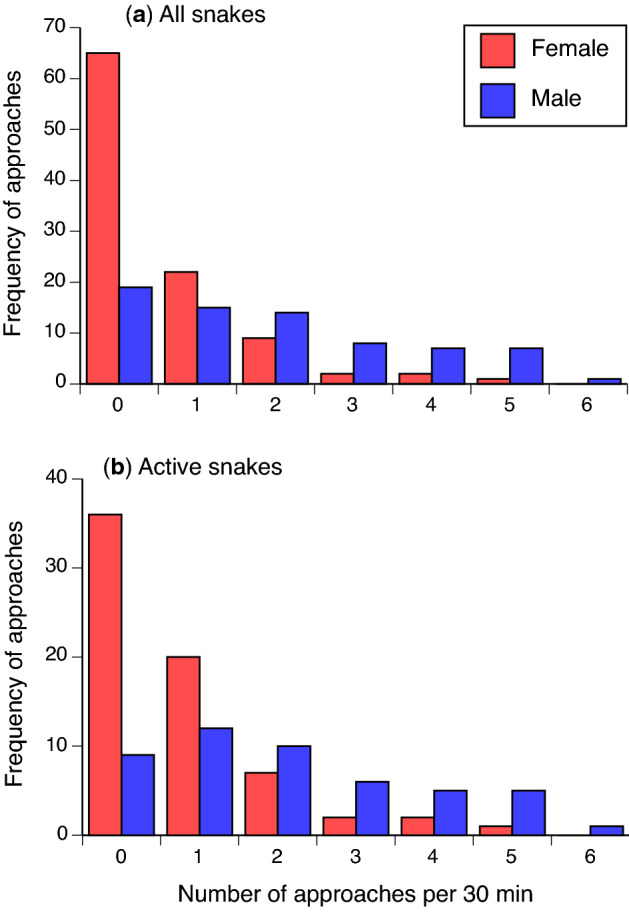
Approaches to divers by Olive sea snakes (*Aipysurus laevis*) as a function of snake sex. The graph shows the total frequency of approaches per 30-min observation period recorded over the duration of the study, divided based on whether all snakes are included (**a**) or only snakes that were active during the focal observation period (**b**).

Male snakes generally approached the diver, whereas few females did so (approaches recorded in 39 of 58 encounters with males vs. 35 of 100 encounters with females; Fisher Exact Test *P* < 0.001; see Fig. 1 and Table 1). Males were rarely observed outside the breeding season (12 of 58). The proportion of females that approached the diver did not differ significantly between breeding and non-breeding seasons (respectively, 10 of 36 encounters vs. 25 of 64 encounters; *P* = 0.28). Removing data for snakes that were inactive did not change these inferences (male vs. female, *P* < 0.001; breeding vs. non-breeding females, *P* = 0.08).

**Table 1:** Sample sizes of Focal Animal Observations (FAO) and Non-Focal Animal Observations (Non-FAO) of Olive sea snakes (*Aipysurus laevis*).

	Males		Breeding season Females		Non-breeding Females	
	FAO	Non-FAO	FAO	Non-FAO	FAO	Non-FAO
Observed	58	16	36	20	64	34
Approached	39	–	10	–	25	–
No approach	19	–	26	–	39	–
Charged diver	1	6	2	4	0	0
Combat	3	10	0	0	0	0
Chase	4	12	0	16	0	0
Coiling	0	3	0	3	0	0

Male snakes were rarely observed outside the breeding season (May–August). In the Table above, “non-breeding females” are those observed outside of the breeding season, whereas “breeding season females” are those observed during the breeding season.

**Figure 2:**
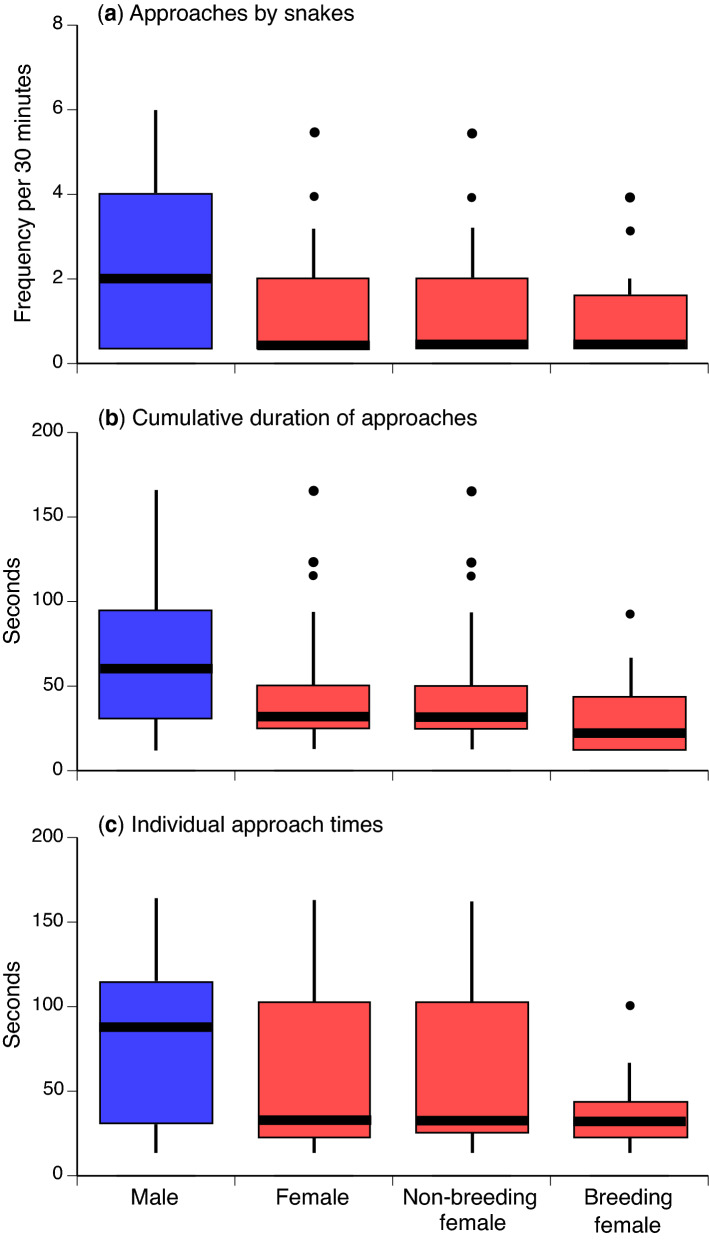
Approach frequency and duration by Olive sea snakes (*Aipysurus laevis*) during focal observation periods. The upper panel (**a**) shows the number of repeat approaches by the same individual within a 30-min focal observation session (data for snakes that did not approach are not included in the lower panels), and the lower panels show the duration of time for which snakes interacted with divers as a function of snake sex and season (breeding vs. non-breeding). Panel (**b**) provides data on cumulative time spent with the diver, whereas panel (**c**) shows mean times per approach. Boldface horizontal line shows median, box shows 25th to 75th percentile, lines are 95% confidence limits, and points show outliers. Focal animal observations: males N = 58, females N = 100, non-breeding season females N = 64, breeding season females N = 36.

Of snakes that approached the diver, males were more likely to do so repeatedly during the 30-min focal observation period, whereas most females only did so once (using data on mean number of approaches per individual [20 males, 34 females] to avoid pseudoreplication: Z = 3.274, *P* < 0.001; Fig. 2a). Hence, males cumulatively spent longer interacting with the diver over the course of the 30-min observation period than did females (using mean values per individual, Z = 3.07, *P* < 0.0025; Fig. 2b). The median time for each individual interaction was also higher for males than for females (excluding one outlier female, Z = 2.99, *P* < 0.003).

For female snakes, the season (breeding vs. non-breeding) did not significantly affect the number of approaches per observation session (Z = 1.312, *P* = 0.19 ; Fig. 2a), the cumulative duration of interactions (Z = 1.42, *P* = 0.15; Fig. 2b) or the mean duration per interaction (Z =  1.06, *P* =  0.29; see Fig. 2c).

Males were also more likely to tongue-flick the diver (proportion of encounters per individual that included tongue-flicking: Z = 2.71, *P* < 0.007). Male snakes typically also tongue-flicked rapidly near the diver’s body, but only 15 males and 2 females actually tongue-flicked the diver’s wetsuit, fins or exposed skin.

Most approaches to divers were slow, and appeared to be investigatory; in contrast, some approaches involved higher speeds and substantial agitation (see Table 1 for sample sizes).

Males chased females during four focal animal observations, and 12 other non-focal males (i.e., males that were not the focus of specific observation periods) were also observed to do so. No focal females were chased (Table 1), reflecting the fact that most observations of females occurred during the non-breeding season when few males were present (above). The duration of the chase was similar in all focal observations, being 150, 160, 120 and 120 s. When chased, females swam fast, changed direction erratically and then entered crevices within the reef. They usually soon re-emerged, using a different exit, and swam above the coral for a short distance before re-entering crevices. In 10 chases the females eluded males by using this tactic. In two cases the females did not reappear, while the males reappeared 15 and 17 min later. On one occasion (23 June 1993), a group of six males chased two females (counted as a single non-focal animal observation). When more than one male chased a female, the males showed no overt reaction to each other.

Charging towards divers only occurred during the breeding season with three focal animals (1 male, 2 females) and 10 non-focal animals (6 males, 4 females) exhibiting this behaviour (Table 1). All charges by males were immediately preceded by either male-male rivalry (apparently, misdirected courtship) or after an unsuccessful chase of a female. For the two female focal animals, one charge occurred after the female had been lost from observation before re-approaching the diver. In the other case, a male was chasing the female. The other four examples of charges by females (i.e., outside focal animal observations) all involved females being chased by males.

Copulation was never observed, but on three occasions non-focal pairs were seen intertwined in a pre-copulatory position. The male positioned himself above and behind the female before quickly throwing two and a half coils around the female to hold her firmly. In each case the coils were positioned along the rear half of the female’s body. In one case the female appeared to be compliant, but the animals disengaged when disturbed by the observer. In the other two cases, the female actively dislodged the male by slithering into a crevice in the coral. Tongue-flicking to the back of the other snake was evident during pre-copulation behaviour.

Female snakes generally ignored each other, but when males sighted each other on the reef they engaged in intense interactions (except for one case after the breeding season had ended, when two males ignored each other). Three male-rivalry focal animal observations were recorded, as were 10 other non-focal rivalry events (Table 1). Duration of the interactions between males was variable, with two long bouts (130 and 135 s) and one brief bout (5 s). In each case, male snakes charged towards each other before pausing in the last 1–2 m of the approach and slowly moving into contact and commencing to tongue-flick each other intensely. This interaction would escalate into an extremely fast and highly excited contest, with each animal apparently trying to position itself above and behind the other. At the conclusion of the interaction one of the males would abruptly turn and swim slowly away.

Male snakes were observed to coil around the diver’s fin, and to strike at their reflections in camera lenses, but no bites to divers were recorded during these interactions. Even after charging, unprovoked bites never occurred during underwater observations. However, snakes readily tried to bite when harassed during capture, or (especially) when handled on the boat after capture. Male snakes can be highly persistent in their attempts to approach divers. On one occasion the diver attempted to flee from a snake by swimming vigorously for 20 min but was unable to outpace his follower. When the diver finally stopped, the snake tongue-flicked him for a minute and then left.

## Discussion

Our data confirm reports that Olive sea snakes often approach divers, and that this behaviour is especially common during the mating season and is exhibited primarily (but not exclusively) by males. Most approaches to divers (especially by females) appeared to have an investigatory purpose; the snake simply attempted to ascertain the nature of the new arrival. A Markovian-chain analysis of these data, to examine probabilities of one behaviour following another, showed that approaches to divers generally occurred when the snakes swam towards the surface to take a breath, and at the beginning of the 30-min observation period^14^. These biases suggest that snakes approached divers as soon as the animal noticed the person.

A smaller subset of animals exhibited “excitable” charges (fast, jerky movements). These behaviours were exhibited primarily by male snakes in the breeding season, and by females that were being pursued by males. We suggest that Olive sea snakes approach divers because of mistaken identity. For example, a reproductively active male, highly aroused, mistakes the diver for another snake (a female or a rival male). At first sight, the idea that a snake might mistake a human diver for another snake seems ludicrous, given the massive disparity in size and shape between those two objects. Nonetheless, this offers the most plausible explanation for our observations.

Terrestrial snakes (including terrestrial elapids, the sister-group to hydrophiines) rely primarily upon substrate-deposited pheromones to locate and recognise females^15^. Such cues are less available in the marine environment, because the female does not crawl along the substrate but rather swims above it. Females hence do not leave a continuous substrate-deposited chemical trail. The sexually-informative molecules are lipids that are not water-soluble and hence, cannot be detected from a distance^16^. As a result, male sea snakes may experience great difficulty in precisely locating females, and in retaining contact with them during courtship^16^.

The transition to aquatic life has resulted in profound changes to visual systems in marine snakes, because light transmission is strongly affected by water^17,18^. Although Olive sea snakes have better visual acuity than some other hydrophiine species^17^, they do not have as much ability in this respect as some terrestrial snakes; and the light-scattering nature of water renders precise visual detection more difficult^19^. Hence, sea snakes may find it difficult to see clearly underwater. Coiling around limbs (otherwise, seen only during courtship) and bites directed towards reflective objects such as camera lenses (present study) and diver face-masks (R. Somaweera, pers. comm.) are consistent with the hypothesis that “attacks” on divers are triggered by a male snake mistaking a diver for a rival snake or potential mate. Males of the Turtle-headed sea snake, *Emydocephalus annulatus*, often court inappropriate objects (such as beche-de-mer) and rapidly approach human divers when contact is lost with a female^16^. These behaviours are almost identical to those we report for *A. laevis*, but the potential consequences for a diver are very different. *Emydocephalus* species are small and non-toxic, whereas *A. laevis* is large and possesses a deadly venom.

Males in several other kinds of marine vertebrates likewise have been reported to target courtship towards people. For example, habituated male dolphins (*Tursiops truncatus*) frequently misdirect courtship behaviour towards humans, and sometimes direct aggressive behaviour to the interlopers^20^. Male dugongs (*Dugong dugon*) have been recorded to do the same (G. Maniel, pers. comm.), as have sea lions (*Otaria flavescens*)^21^. Male sea turtles (*Chelonia mydas*) at breeding beaches try to copulate with scuba divers, and can be difficult to dislodge^22^. Similar examples of males using inappropriate cues to identify sexual partners abound in terrestrial systems as well; for example, some beetles famously court beer bottles (the buprestid beetle *Julodiorpha bakewelli*)^23^.

More generally, snakes sometimes fail to distinguish humans from other taxa that to our eyes, look very different. For example, snakes occasionally make unprovoked foraging attacks on people who are far too large to ingest^24^. A reliance upon epidermal chemical cues to assess a conspecific’s gender and reproductive state^25^ means that an amorous male snake has to approach its target close enough to tongue-flick the skin; and hence, visual cues to an intruder’s size and identity may be ignored in favour of chemical evidence obtainable only through direct contact. Although our study provides the first quantitative evidence on sea snake “attacks”, the patterns that we observe accord well with earlier statements. Thus, for example, Udyawer et al.^6^ report that male Greater sea snakes (*Hydrophis major*) approach and interact with humans during the breeding season, and Heatwole^8^ describes cases of reproductively active male snakes (including, *A. laevis*) “charging” a diver.

The other common situation in which agitated snakes swam rapidly towards divers was when a female attempted to flee from courting males. In these cases, the diver likely was perceived as a potential hiding place. Again, both sexes of *E. annulatus* also frequently approach human divers in this way, to escape from harassment by humans (R. Shine, pers. obs.). In the course of mark-recapture work on *E. annulatus*^26^, we often catch a snake underwater, examine it and then release it. The snakes then swim off rapidly, and usually disappear into a crevice in the coral—but a snake that cannot locate a suitable crevice may turn and race back to the diver, and try to hide beside him or her. Intuition suggests that the snake mistakes the diver for a coral tower that would offer refuge.

Other “attacks” may be the result of interference by humans; a snake that has been handled or struck may retaliate vigorously, and be difficult to discourage^8^. If mistaken identity underlies most “attacks” by sea snakes on divers, the best strategy for divers in such a situation may be to allow the snake to investigate them and in particular to allow for the snake to investigate chemical cues with its tongue; a bite is unlikely unless the animal is threatened or injured (as also advocated by Heatwole^8^). Attempting to flee is likely to be futile and may even increase the ardour of the pursuit; and attempting to drive the animal away may induce retaliation.

## Materials and methods

### Study species

**Figure 3:**
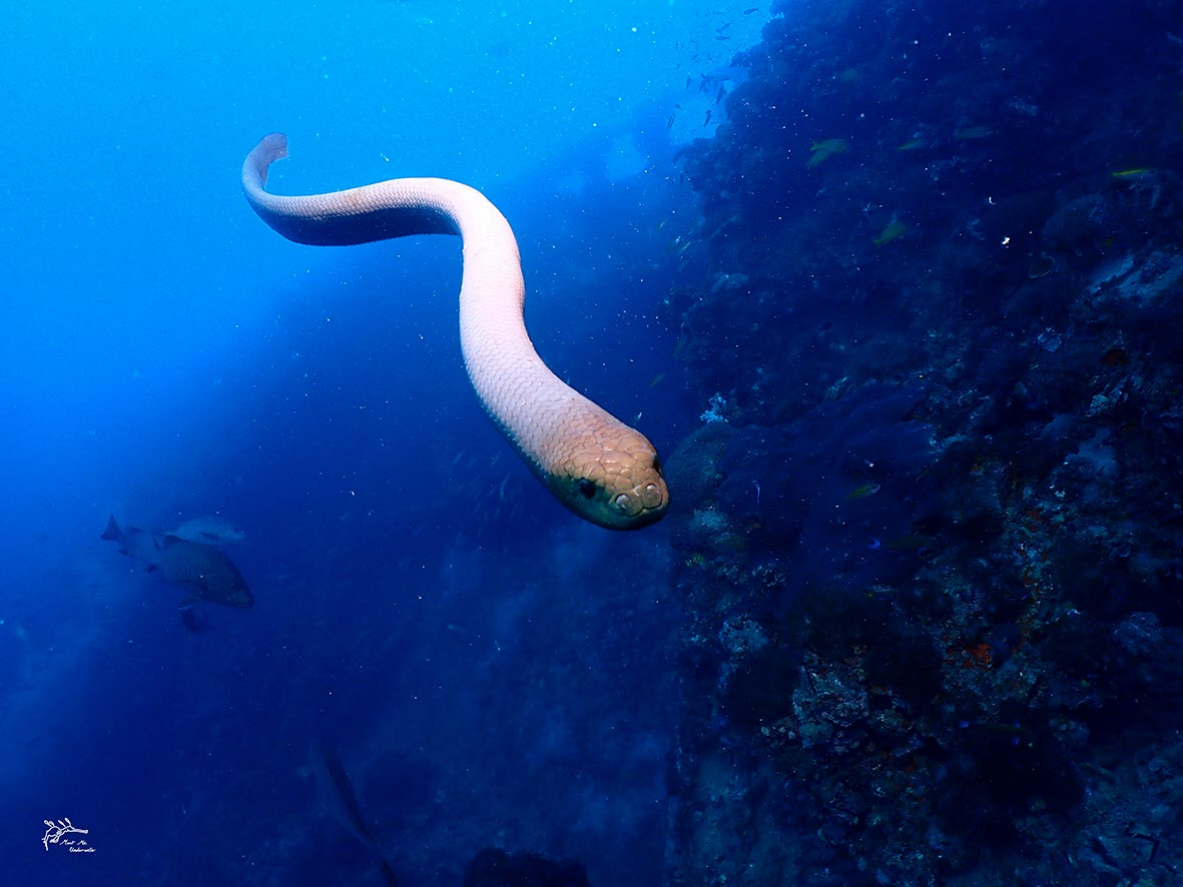
A male Olive sea snake, *Aipysurus laevis*, swimming directly towards a camera held by a diver. Photograph by Jack Breedon, with permission.

*Aipysurus laevis* is a large and heavy-bodied sea snake species, with adult females sometimes exceeding 2 m in length and 3 kg in mass (see Fig. 3); males are smaller (usually 500 g but up to 700 g at our study site)^14^. The species is widely distributed through the oceans of tropical Australia and New Guinea^27^. Females occupy reef habitats year-round, but adult males are present at reefs primarily during the breeding season (winter: May to August)^14,28^. At this time, males spend much of their time swimming rapidly along the reef edge and courting any females they encounter. Females often flee from these courtship attempts, by taking refuge within coral crevices or swimming away rapidly^14^. The species has a broad diet that includes snails, crustaceans and fish^2,29^.

### Study area

Passage Rocks (23° 10′ S, 150° 57′ E) lies 20 km off the east coast of Australia, within the Keppel Group of continental islands, and inside the southern section of the Great Barrier Reef. Water temperatures average 27.9 °C in midsummer (February) and 20.7 °C in midwinter (August: https://www.seatemperature.org/australia-pacific/australia/yeppoon). The substrate comprises a mosaic of boulders, various types of coral, seagrass, and sandy areas; female *A. laevis* often use shallow reef-crest habitats whereas males are more often found along the edges of the reef, in deeper water^14^.

### Methods

One of us (TPL) SCUBA-dived across 188 dives (total of 15,040 min) to conduct Focal Animal Observations (FAO), generally of 30-min duration, over the period May 1994 to July 1995^14^. This resulted in a dataset of 158 observations of snakes. TPL recorded the number of snakes that approached him, and the frequency and duration of those approaches within each 30-min period. To standardise interactions between the observing diver and snakes, TPL responded to the approach of a snake by settling onto the seafloor and remaining immobile until the snake moved away. The depth of the seafloor varied considerably throughout the study area, but was not linked in any obvious way to the likelihood of a snake approaching the diver.

Data were also collected on the snake’s sex and the number and duration of approaches and sexual behaviours. In this species, sex of adult snakes can be accurately determined by body size (above) but also by colour (females are blue-grey, males are brown) and scale rugosity (females have smooth scales, males are rugose)^14,28^. Behaviours were categorised into: chasing of females by males, coiling between snakes (courtship), male-male rivalry, charging the diver and/or approaching the diver. The duration in seconds of each approach by focal animal snakes was also recorded. Most approaches to the diver were slow, with rapid approaches (“charges”) infrequent. In addition to observations of focal animals, data on sexual behaviour were collected on other snakes but as counts only. Two final behaviours, resting and tongue-flicking were also quantified. Snakes were categorised as resting if they were inactive under the coral and did not emerge over the 30-min observation period, or if they transferred to this behaviour during the first 2 min of observation. Tongue-flicking of the diver by snakes was recorded during encounters.

Approaches by snakes towards divers were divided into four categories: those by male snakes (which were mostly present on the reef during the breeding season [May–August]), all female snakes, female snakes during the breeding season, and those by female snakes outside the breeding season (Sept-April). Relative number of approaches, tongue-flicking, cumulative time of approach across the focal animal observation and also individual durations of encounters by category were compared non-parametrically using Fisher’s Exact Test and the Wilcoxon test with JMP Pro 15. These analyses used only data for snakes of known ID, to avoid pseudoreplication (i.e., each snake contributed only a single mean value to each analysis). To explore if season (breeding vs. non-breeding) affected approach behaviour of female snakes, we treated data for each snake in each season as independent (a procedure that did not affect our conclusions by spuriously inflating significance, because all comparisons were non-significant).

All procedures were designed to minimize stress to the animals, were carried out in accordance with relevant guidelines and regulations (including ARRIVE guidelines), and were approved by the James Cook University animal care and ethics committee, and by Queensland National Parks and Wildlife Service. Funding was provided by the Lion’s Club of Townsville.

## Data Availability

Data have been deposited in Dryad DOI 10.5061/dryad.xwdbrv1dq
